# Comparative Clinical and Imaging‐Based Evaluation of Therapeutic Modalities in CNS Embryonal Tumours With PLAGL Amplification

**DOI:** 10.1111/nan.70015

**Published:** 2025-04-08

**Authors:** Michaela‐Kristina Keck, Anna Tietze, Brigitte Bison, Shivaram Avula, Julien Engelhardt, Cécile Faure‐Conter, Tanguy Fenouil, Dominique Figarella‐Branger, Einar Goebell, Johannes Gojo, Christine Haberler, Juhana Hakumäki, James T. Hayden, Laura S. Korhonen, Ewa Koscielniak, Christof M. Kramm, Mariëtte E. G. Kranendonk, Maarten Lequin, Louise E. Ludlow, David Meyronet, Per Nyman, Ingrid Øra, Thomas Perwein, Jouni Pesola, Tuomas Rauramaa, Roel Reddingius, David Samuel, Antoinette Y. N. Schouten‐van Meeteren, Alexandra Sexton‐Oates, Alexandre Vasiljevic, Thekla von Kalle, Annika K. Wefers, Pieter Wesseling, Josef Zamecnik, Michal Zapotocky, Katja von Hoff, David T. W. Jones

**Affiliations:** ^1^ Division of Pediatric Glioma Research Hopp Children's Cancer Center Heidelberg (KiTZ) Heidelberg Germany; ^2^ National Center for Tumor Diseases (NCT), NCT Heidelberg A Partnership Between DKFZ and Heidelberg University Hospital Heidelberg Germany; ^3^ German Cancer Research Center (DKFZ) Heidelberg Germany; ^4^ Institute of Neuroradiology Charité—Universitätsmedizin Berlin, corporate member of Freie Universität Berlin and Humboldt‐Universität zu Berlin Berlin Germany; ^5^ Department of Neuroradiology University Hospital Augsburg Augsburg Germany; ^6^ Department of Radiology Alder Hey Children's NHS Foundation Trust Liverpool UK; ^7^ Service de Neurochirurgie B CHU de Bordeaux Bordeaux France; ^8^ Université de Bordeaux, Bordeaux INP, CNRS, IMB, UMR 5251 Talence France; ^9^ Institut d'Hemato‐oncologie Pediatrique Lyon France; ^10^ Institut de Pathologie Est, Hospices Civils de Lyon Université Claude Bernard Lyon 1, INSERM 1052, CNRS 5286, Centre de Recherche en Cancérologie de Lyon Lyon France; ^11^ Aix‐Marseille University, APHM, CNRS, INP, Institute De Neurophysiopathologie, CHU Timone Service d'Anatomie Pathologique et de Neuropathologie Marseille France; ^12^ Department of Diagnostic and Interventional Neuroradiology University Medical Center Hamburg‐Eppendorf Hamburg Germany; ^13^ Department of Pediatrics and Adolescent Medicine, Comprehensive Cancer Center and Comprehensive Center for Pediatrics Medical University of Vienna Vienna Austria; ^14^ Division of Neuropathology and Neurochemistry, Department of Neurology Medical University of Vienna Vienna Austria; ^15^ Department of Clinical Radiology Kuopio University Hospital Kuopio Finland; ^16^ Institute of Clinical Medicine University of Eastern Finland Kuopio Finland; ^17^ Department of Pediatric Hematology and Oncology Alder Hey Children's NHS Foundation Trust Liverpool UK; ^18^ Department of Pediatrics and Adolescent Medicine Turku University Hospital and University of Turku Turku Finland; ^19^ Department of Pediatric Oncology/Hematology/Immunology Olgahospital, Klinikum Stuttgart Stuttgart Germany; ^20^ Division of Pediatric Hematology and Oncology University Medical Center Göttingen Göttingen Germany; ^21^ Princess Máxima Center for Pediatric Oncology Utrecht The Netherlands; ^22^ Division of Imaging and Oncology, Department of Radiology UMC Utrecht the Netherlands; ^23^ Murdoch Children's Research Institute The Royal Children's Hospital Parkville Victoria Australia; ^24^ Children's Cancer Centre The Royal Children's Hospital Parkville Victoria Australia; ^25^ Department of Paediatrics The University of Melbourne Parkville Victoria Australia; ^26^ Crown Princess Victoria Children's Hospital Linköping University Hospital Linköping Sweden; ^27^ Department of Biomedical and Clinical Sciences Linköping University Linköping Sweden; ^28^ Department of Pediatric Oncology and Hematology, Skåne University Hospital Lund University Lund Sweden; ^29^ Division of Pediatric Hemato‐Oncology, Department of Pediatrics and Adolescent Medicine Medical University of Graz Graz Austria; ^30^ Styrian Children's Cancer Research—Research Unit for Cancer and Inborn Errors of the Blood and Immunity in Children Medical University of Graz Graz Austria; ^31^ Department of Pediatrics, Pediatric Hematology and Oncology Ward, Kuopio University Hospital and Institute of Clinical Medicine University of Eastern Finland Kuopio Finland; ^32^ Department of Clinical Pathology, Kuopio University Hospital and Unit of Pathology, Institute of Clinical Medicine University of Eastern Finland Kuopio Finland; ^33^ Department of Neuro‐Oncology Princess Máxima Center for Pediatric Oncology Utrecht the Netherlands; ^34^ Department of Pediatric Hematology‐Oncology Valley Children's Hospital Madera California USA; ^35^ Rare Cancers Genomics Team, Genomic Epidemiology Branch International Agency for Research on Cancer/World Health Organization Lyon France; ^36^ Department of Radiology Olgahospital, Klinikum Stuttgart Stuttgart Germany; ^37^ Institute of Neuropathology University Medical Center Hamburg‐Eppendorf Hamburg Germany; ^38^ Mildred Scheel Cancer Career Center HaTriCS4 University Medical Center Hamburg‐Eppendorf Hamburg Germany; ^39^ Department of Pathology Amsterdam University Medical Centers, Location VUmc and Brain Tumor Center Amsterdam Amsterdam the Netherlands; ^40^ Department of Pathology and Molecular Medicine, Second Faculty of Medicine Charles University and University Hospital Motol Prague Czech Republic; ^41^ Prague Brain Tumor Research Group, Second Faculty of Medicine Charles University and University Hospital Motol Prague Czech Republic; ^42^ Department of Pediatric Haematology and Oncology, Second Faculty of Medicine Charles University and University Hospital Motol Prague Czech Republic; ^43^ Department of Pediatric Oncology and Hematology Charité—Universitätsmedizin Berlin, Corporate Member of Freie Universität Berlin, Humboldt‐Universität zu Berlin, and Berlin Institute of Health Berlin Germany; ^44^ Department of Paediatric and Adolescent Medicine Aarhus University Hospital Aarhus Denmark

**Keywords:** embryonal CNS tumour, ET, PLAGL, *PLAGL1*, *PLAGL2*, treatment

## Abstract

**Aims:**

Embryonal tumours with *PLAGL1* or *PLAGL2* amplification (ET, PLAGL) show substantial heterogeneity regarding their clinical characteristics and have been treated inconsistently, resulting in diverse outcomes. In this study, we aimed to evaluate the clinical behaviour of ET, PLAGL and elucidate their response pattern across the different applied treatment regimens.

**Methods:**

We conducted an in‐depth retrospective analysis of clinical and serial imaging data of 18 patients with ET, PLAGL (nine each of *PLAGL1* and *PLAGL2* amplified).

**Results:**

Patients with *PLAGL1*‐amplified tumours (ET, PLAGL1) had fewer relapses (3/9), while *PLAGL2*‐amplified tumours (ET, PLAGL2) were prone to early relapse or progression (8/9) and to distant, leptomeningeal and intraventricular relapses. Progression‐free survival differed significantly between the subtypes (log‐rank test, *p* = 0.0055). Postoperative treatment included chemotherapy (*n* = 17, various protocols), alone (*n* = 8) or combined with radiotherapy (*n* = 9). Responses to chemotherapy were observed in both subtypes, and incomplete resection was not associated with inferior survival. All three survivors with ET, PLAGL2 were treated with induction and high‐dose chemotherapy with (*n* = 1—low‐dose CSI and boost) or without (*n* = 2) radiotherapy, whereas five patients with less intensive chemotherapy relapsed. All six survivors with ET, PLAGL1 were treated with conventional chemotherapy regimens, with (*n* = 4—local radiotherapy *n* = 3; CSI and boost *n* = 1) or without (*n* = 2) radiotherapy. Two patients with ET, PLAGL1 relapsed after 8 years.

**Conclusions:**

Adjuvant therapy should be considered for all ET, PLAGL patients: Patients with ET, PLAGL2 might benefit from intensified chemotherapy regimens. In contrast, patients with ET, PLAGL1 showed superior outcomes without high‐dose chemotherapy or craniospinal irradiation.

**Summary**

- Embryonal tumours with *PLAGL1* or *PLAGL2* amplification are two (epi)genetically different subtypes, whose differences in clinical behaviour are understudied.
- To date, optimal treatment strategies remain elusive for both subtypes.
- Objective responses to chemotherapy were observed in patients with *PLAGL1*‐ and *PLAGL2*‐amplified tumours.
- *PLAGL1*‐amplified tumours showed relatively favourable progression‐free survival and a trend towards better overall survival, were more often sufficiently treated with less intensive treatment regimens with or without radiotherapy, but showed late recurrences.
- *PLAGL2*‐amplified tumours often displayed early relapses, in particular at distant sites, scrutinising the application of local radiotherapy. Relapses were more frequent among patients with less intensive treatment regimens as compared to patients treated with intensified chemotherapy.

## 1 Introduction

Paediatric central nervous system (CNS) tumours show substantial molecular, histopathological and clinical heterogeneity. Over recent years, it has become evident that paediatric CNS tumours can be classified and distinguished based on their methylation profiles and other molecular features [1, 2, 3, 4, 5, 6, 7, 8]. This concept is also reflected in the latest (2021) edition of the World Health Organization (WHO) Classification of CNS Tumours, which recognises a wide range of paediatric CNS tumour types and subtypes [4]. As methylation data accumulates, the molecular classification based on DNA methylation profiling is continuously refined and novel rare CNS tumour types can now be discriminated [9, 10, 11]. Both molecular and clinical characterisation of these newly delineated tumour types is required to elucidate rational treatment options with established treatment regimens or novel, personalised strategies. One of the recently defined novel paediatric tumour types (not yet included in the WHO classification of CNS tumours) is termed CNS embryonal tumour with PLAGL amplification (ET, PLAGL), which is marked by amplification of either *PLAGL1* or *PLAGL2* [10]—subsequently referred to as ET, PLAGL1 and ET, PLAGL2, respectively. ET, PLAGL show substantial variation in anatomical location, clinical presentation and histological morphology. As a result, they have previously been treated with heterogeneous regimens. Slightly diverging methylation profiles of ET, PLAGL1 and ET, PLAGL2 were reported previously, indicative of the existence of two subtypes that differ in their age distribution (median age at diagnosis 10.5 and 2 years for ET, PLAGL1 and ET, PLAGL2, respectively) and appear to have differing prognoses [10]. Using methylation profiling, we can now reliably diagnose ET, PLAGL, but there is a lack of clinical and therapeutic evidence, and selecting treatment strategies remains a challenge. Our study focuses on describing the clinical behaviour and treatment response of *PLAGL1/2*‐amplified tumours, intending to inform treatment decisions and future clinical research collaborations on this rare tumour type for which no treatment standard is available.

To address this, we analysed a comprehensive set of clinical and MRI data from 18 patients with ET, PLAGL (nine ET, PLAGL1 and nine ET, PLAGL2) evaluating the treatment response, relapse pattern and survival, descriptively comparing the different treatment regimens to inform treatment decisions for this clinically largely unknown type.

## 2 Materials and Methods

### 2.1 Molecular Classification

Classification of tumours as ET, PLAGL through genome‐wide DNA methylation profiling, *t*‐distributed stochastic neighbour embedding (*t*‐SNE) dimensionality reduction and copy number variation (CNV) analysis based on the raw intensities of the methylation array probes was performed as described in Keck et al. [10] using the methodology described in [1, 12].

### 2.2 Cohort Composition

Clinical data and serial MR data from patients with confirmed ET, PLAGL were requested from international paediatric oncology centres and institutions. The pretreatment and post‐treatment imaging data were provided either directly by the respective local participating centre or by the national radiology reference centre, according to the patient/parental consent and local ethics approval. Eighteen patients from 14 centres with complete clinical information and available MRI scans were included in the analysis. The majority of these patients (*n* = 15) were already part of a previous publication on ET, PLAGL [10], while three further patients classified as ET, PLAGL and with confirmed *PLAGL1* or *PLAGL2* amplification were added to the current cohort.

Six of the 21 ET, PLAGL patients with clinical documentation referenced in [10] were not included in this cohort due to incomplete clinical information and unavailability of MRI scans, but the available clinical information for these patients is summarised in Table 1.

**TABLE 1:** Cases with incomplete clinical documentation and no available MRI scans. Thirty‐one ET, PLAGL cases were presented in Keck et al. [10]; 21 of these cases had clinical documentation and amplification of *PLAGL1* or *PLAGL2*, 15 of which are part of our current study. Listed are the six cases that dropped out of our current cohort due to incomplete clinical documentation or unavailability of MRI scans.

ID	Amp	Age	Treatment	OS (years)	Status at last follow‐up	Relapse	Not available
A93	PLAGL1	4–18	GTR, CT, CSI with boost	15	Alive	No relapse, no known metastasis, but no CSF work done	Type of CT, dosage of RT, duration of treatment, MRI
A388	PLAGL1	4–18	GTR, no CT, RT	0.4	Dead	NA	Type and dosage of RT, MRI
A106	PLAGL2	0–3	NA	4.7	Alive	NA	Treatment data, MRI
A108	PLAGL2	0–3	Resection, CT, RT	3.4	Alive	No relapse, no metastasis	Extent of surgery, type of CT, type and dosage of RT, duration of treatment, MRI
A94	PLAGL2	4–18	NA	0.7	Alive	No relapse	Treatment data, MRI
A110	PLAGL2	0–3	GTR, CT and ASCR (incl. carboplatin and thiotepa), no RT	2.25	Alive	No relapse, CR	Duration of treatment, MRI

Abbreviations: Amp, amplification; NA, not available.

### 2.3 Survival Analysis and Statistical Analyses

Survival analyses were performed using R version 4.2.2 [13]. The Kaplan–Meier method was used to determine overall survival (OS) and progression‐free survival (PFS), and the log‐rank test (*p* value) was applied to identify differences between the Kaplan–Meier curves. OS was defined as the time between the first diagnosis and the last follow‐up date or death, and PFS was defined as the time between the first diagnosis and the time point of the first relapse or progression. A comprehensive summary of survival times, treatments and outcomes is presented as a swimmer's plot. Different associations between type of amplification, sex, location, survival and resection status were computed using Fisher's exact test and GraphPad Prism version 10.2.1 for Windows (GraphPad Software, Boston, Massachusetts, USA, www.graphpad.com).

### 2.4 MRI Analysis

MRI analysis was performed by two experienced paediatric neuroradiologists (A.T. and B.B.) employing well‐established MRI criteria [14] as described in Tietze et al. [15]. The initial MRI scans were assessed through consensus decisions of both, whereas subsequent follow‐up MRI data were evaluated independently using the same criteria. The evaluation of response adhered to the guidelines set forth by the European Society for Paediatric Oncology (SIOPE) Brain Tumour Group [16].

## 3 Results

### 3.1 Clinical Characteristics of ET, PLAGL1 and ET, PLAGL2 Differed

**FIGURE 1:**
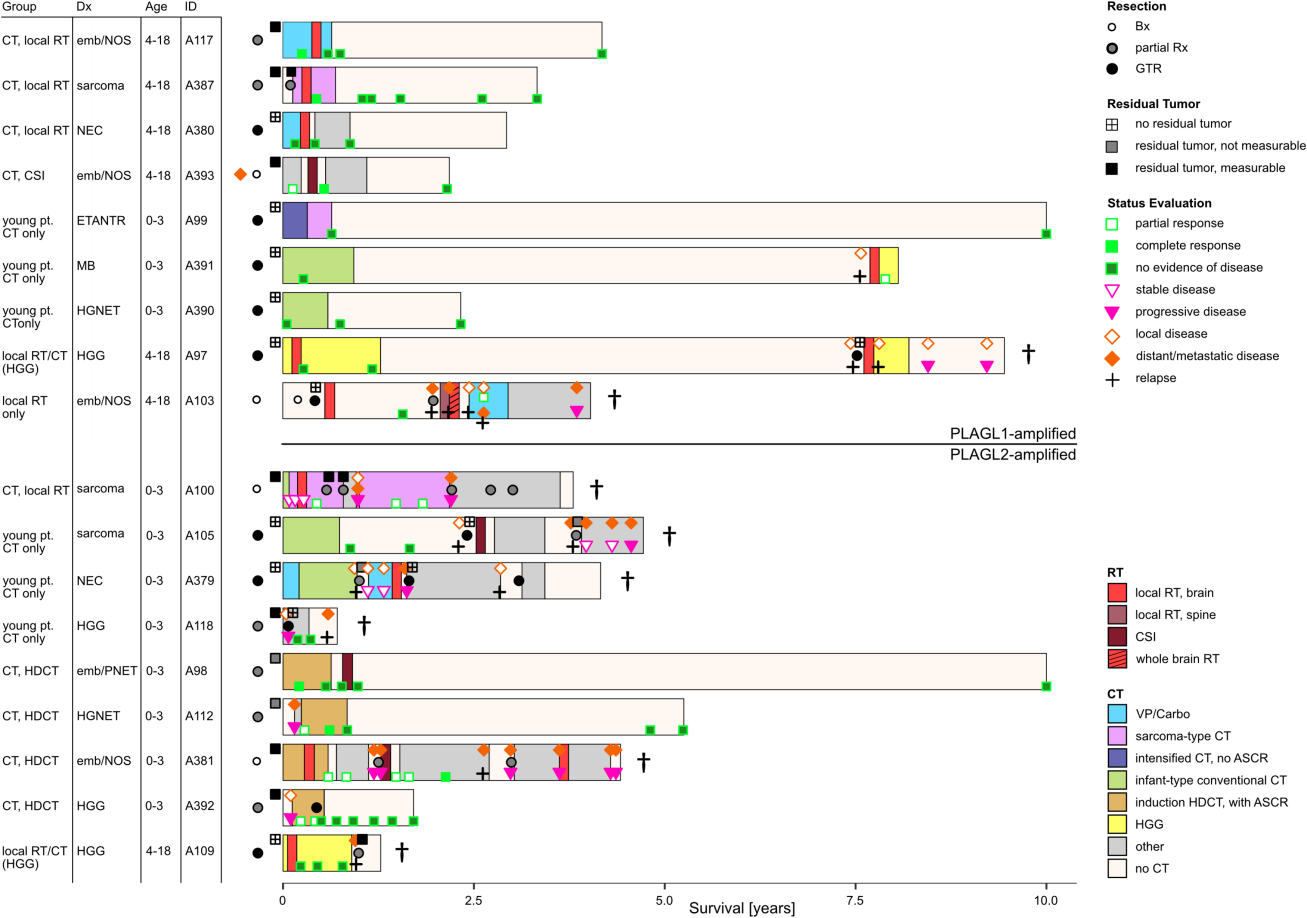
Detailed summary of clinical information and outcome of all 18 patients separated according to amplification status, *PLAGL1* (*n* = 9) and *PLAGL2* (*n* = 9). OS is shown on the *x* axis. IDs and clinical data are listed for each patient on the left. Bars are coloured according to treatment (CT and RT). The extent of resection, amount of residual tumour after resection and response to treatment were determined through a review of MRI scans and displayed through the different symbols. The death of a patient is symbolised by an obelisk behind the respective bar. ASCR, autologous stem cell rescue; Bx, biopsy; Carbo, carboplatin; CSI, craniospinal irradiation; CT, chemotherapy; Dx, diagnosis; emb, embryonal; ETANTR, embryonal tumour with abundant neuropil and true rosettes; GTR, gross total resection; HDCT, high‐dose chemotherapy; HGG, high‐grade glioma; HGNET, high‐grade neuroepithelial tumour; MB, medulloblastoma; NEC, not elsewhere classified; NOS, not otherwise specified; PNET, primitive neuroectodermal tumour; pt., patient; RT, radiotherapy; Rx, resection; VP, VePesid.

Our cohort comprises 18 clinically annotated patients with CNS tumours classified as ET, PLAGL based on methylation profiling that show amplification of either *PLAGL1* (*n* = 9) or *PLAGL2* (*n* = 9). Female patients were more frequent in ET, PLAGL1 (F:M 6:3) and underrepresented in ET, PLAGL2 (F:M 3:6) (not significant, Fisher's exact test, *p* = 0.3469). The age at diagnosis ranged from 1.4 to 18.3 years for ET, PLAGL1 (median = 8.0 years) and from 1.0 to 5.0 years for ET, PLAGL2 (median = 1.9 years). Primary diagnoses before the establishment of a molecular diagnosis of ET, PLAGL comprised medulloblastoma (*n* = 1), high‐grade glioma (HGG) (*n* = 4), sarcoma (*n* = 3), ETANTR (*n* = 1), PNET (*n* = 1), other embryonal (*n* = 4), neuroepithelial (*n* = 2) or not classified tumours (*n* = 2) (Figure 1).

### 3.2 ET, PLAGL Tumours Arose in a Variety of Brain Structures

**FIGURE 2:**
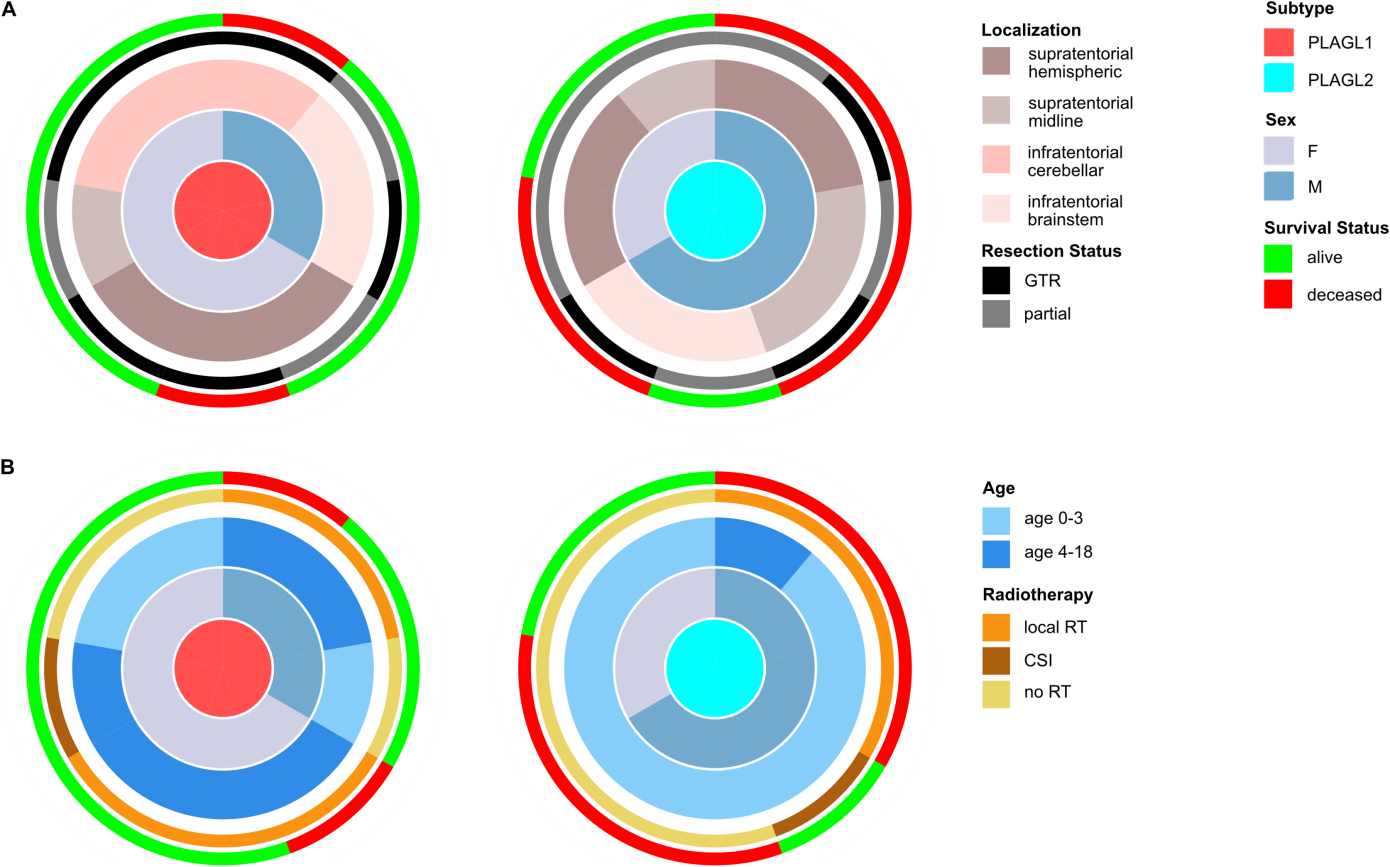
Separate sunburst plots are shown for ET, PLAGL1 and ET, PLAGL2. (A) Localisation of the tumours is shown in conjunction with resection status and survival for male and female patients. (B) Age is shown in conjunction with RT and survival for male and female patients.

The distribution of supratentorial and infratentorial tumours (S:I) was balanced for patients with ET, PLAGL1 (S:I 4:5), while ET, PLAGL2 were slightly more common in the supratentorial compartment (S:I 7:2) (not significant, Fisher's exact test, *p* = 0.3348) (Figure 2A and Table S2). Of the nine ET, PLAGL1, one was located supratentorially in the midline, two in the brainstem, three in the cerebellum and three in the cerebral hemispheres. In contrast, five of the nine ET, PLAGL2 were located in midline structures, three supratentorial and two in the brainstem. The four remaining ET, PLAGL2 were supratentorial with a hemispheric location. One patient in our cohort (ET, PLAGL1) presented with disseminated cerebral disease (no spinal metastases) at the time of diagnosis, and one further patient (ET, PLAGL2) developed an early pretreatment cerebral metastatic relapse (Figure 1 and Table S2).

### 3.3 Resection Status Was Not Associated With Tumour Location

Complete macroscopic resection was achieved before the start of therapy in six patients with ET, PLAGL1 and three patients with ET, PLAGL2, without an association with the type of amplification or infratentorial/supratentorial tumour location (Fisher's exact test, *p* = 0.3469 and 0.3348, respectively) (Figures 1 and 2A). The remaining three patients with ET, PLAGL1 started treatment with measurable residual tumours, two after a partial resection and one after a biopsy only. Of the six patients with incompletely resected ET, PLAGL2, residual tumour was not measurable in two patients, and four patients had measurable residual tumour—two after biopsy and two after a partial resection (Figure 1). Three ET, PLAGL2 patients with postoperative residual tumours experienced early progression before or shortly after the onset of treatment, two with local progression and one patient with an intraventricular tumour developed intraventricular metastases (Table S3).

### 3.4 Applied Treatment Regimens Were Heterogeneous and Differed Between the Two Subtypes

Corresponding to the various initial diagnoses and based on age at diagnosis, a broad range of oncological treatment regimens were applied (Figure 1 and Tables S1 and S4). All but one patient received chemotherapy as part of their primary treatment, including different infant‐type conventional chemotherapy regimens (two ET, PLAGL1 and three ET, PLAGL2), sarcoma‐type chemotherapy (two ET, PLAGL1 and one ET, PLAGL2), intensified induction chemotherapy (one ET, PLAGL1), induction and high‐dose chemotherapy with autologous stem cell rescue (HDCT/ASCR) (four ET, PLAGL2) and HGG regimen with temozolomide (one ET, PLAGL1/2, each). Other chemotherapy treatments used carboplatin/etoposide combination (two ET, PLAGL1 and one ET, PLAGL2) or other agents. Further various treatment regimens were applied at relapse (Tables S1 and S4).

### 3.5 Favourable Responses to Chemotherapy Were Achieved for ET, PLAGL1 With Less Intensive Treatment

All eight ET, PLAGL1 patients that were treated with chemotherapy in the primary treatment received conventional chemotherapy regimens without the use of HDCT/ASCR. Five of these eight patients had a gross total resection and remained in remission throughout treatment and beyond, with (*n* = 2) or without (*n* = 3) the use of additional radiotherapy in the primary treatment. Two of the five patients developed a late local relapse (one each with/without radiotherapy in the primary treatment) (see below and Figure 1).

All three patients with incompletely resected ET, PLAGL1 achieved complete remission during initial therapy (Figures 1 and 2A). A radiological response to chemotherapy was observed in 2/2 patients with available MRI imaging before the onset of radiotherapy: A complete response was observed in a patient who had a localised tumour and was treated with carboplatin/etoposide before proceeding to local radiotherapy. A partial response was observed in a patient who had initial metastatic disease and a biopsy, after five PEI courses (cisplatin, etoposide and ifosfamide) with intraventricular medication (alternating etoposide, cytarabine, topotecan). This patient went on to achieve a complete remission after subsequent radiotherapy (36‐Gy craniospinal irradiation (CSI)/54‐Gy boost). For the third patient, a complete remission was observed after combined treatment with a (CNS‐)sarcoma‐type chemotherapy (ICE, ifosfamide, carboplatin and etoposide) and local irradiation (Figure 1 and Tables S1 and S4). To date, all three patients are alive with no evidence of disease for 4, 2 and 3 years after diagnosis, respectively.

All nine patients with ET, PLAGL2 received chemotherapy in the primary treatment, five with conventional chemotherapy regimens and four with induction/HDCT/ASCR. All three patients who had a macroscopic complete resection at the initial surgery were treated with conventional chemotherapy regimens with (*n* = 1) or without (*n* = 2) radiotherapy, relapsed early, 12–28 months after the diagnosis and succumbed to their disease. Of six patients with incompletely resected ET, PLAGL2, four achieved complete remission on primary treatment. One patient achieved this by macroscopic complete re‐resection of the local progressive disease but relapsed shortly after the end of the conventional chemotherapy treatment. Three radiotherapy‐naïve patients showed a radiological response to chemotherapy with induction/HDCT/ASCR. This included one patient with pretreatment distant progression and one patient with pretreatment local progression who showed a partial response on induction/HDCT/ASCR and achieved complete remission after re‐resection of the local residual tumour. The latter two patients did not receive radiotherapy in the further course. The third patient achieved a complete response following induction/HDCT and subsequently received CSI. At the last follow‐up, all three patients were in complete first remission, 20 months, 5 years and 15 years after diagnosis.

Two patients with incompletely resected ET, PLAGL2 achieved only a partial response. One patient was treated with induction chemotherapy, subsequent local radiotherapy and HDCT/ASCR thereafter, and showed a partial response at the end of the initial treatment. This prompted additional chemotherapy, but the patient subsequently experienced progression at a distant cerebral site. One patient had stable disease after infant‐type and sarcoma‐type conventional chemotherapy showed a partial response after subsequent local radiotherapy but did not achieve complete remission during primary treatment with additional sarcoma‐type chemotherapy (Figure 1).

### 3.6 Various Radiotherapy Strategies Were Applied

Depending on the patient's age, the clinical staging and the choice of treatment protocol, different radiotherapy regimens were selected (Figures 1 and 2B and Table S4).

An infant‐type, radiotherapy‐omitting regimen was chosen for three patients with ET, PLAGL1 (1.4–3.0 years of age at diagnosis) and five patients with ET, PLAGL2 (1.0–2.1 years at diagnosis). Overall, four of these eight patients were alive and in complete remission at the last follow‐up (two ET, PLAGL1 and two ET, PLAGL2).

Three patients younger than 4 years at diagnosis (all three ET, PLAGL2) and all seven patients older than 4 years at diagnosis (six ET, PLAGL1 and one ET, PLAGL2) received radiotherapy within the primary treatment. Of these 10 patients, eight received local radiotherapy to the tumour bed (five ET, PLAGL1 and three ET, PLAGL2), and two patients received CSI/boost. The latter included one patient with ET, PLAGL1 with initial leptomeningeal metastatic presentation and one patient with a localised ET, PLAGL2 who received low‐dose CSI/boost (19.4/54 Gy) at the age of 3 years, after HDCT/ASCR treatment. Overall, three of eight patients with local radiotherapy (all ET, PLAGL1) and two of two patients with CSI/boost were alive in complete remission at the last follow‐up (Figures 1 and 2B).

### 3.7 More Frequent and More Distant Relapses Were Observed in Patients With ET, PLAGL2

Overall, 3/9 patients with ET, PLAGL1 and 8/9 patients with ET, PLAGL2 experienced relapse or progression.

Within the ET, PLAGL1‐group, one patient experienced a distant relapse 2 years post diagnosis, while two others suffered local relapses after 8 years. The patient with the distant relapse was the only patient in the ET, PLAGL1 group that was treated with resection and local radiotherapy only, that is, without chemotherapy. He developed a distant leptomeningeal relapse in the lumbar part of the spine 15 months after initial diagnosis, with a particularly complex multimetastatic, intracranial and spinal leptomeningeal relapse pattern, thereafter, leading to the patient's death, 2.1 years after diagnosis of the first relapse. The other two patients with relapses were initially treated with complete macroscopic resection and subsequent conventional infant‐type CT without radiotherapy and HGG therapy with local irradiation and temozolomide, respectively. Both patients experienced a local relapse 8 years post diagnosis. The molecular diagnosis of ET, PLAGL1 was confirmed, and treatment with local radiotherapy and temozolomide was used for both patients with late relapses.

To date, six patients with ET, PLAGL1 are in first complete remission, 2–11 years after diagnosis. All six patients were treated with various conventional chemotherapy protocols, and 4/6 patients received radiotherapy (three local/one CSI), as described above.

Notably, from the additional cohort of patients with incomplete clinical data, a further patient with ET, PLAGL1 that received radiotherapy only, succumbed to the tumour within 5 months after diagnosis (A388), while a second patient treated with CT and CSI/boost is a long‐term survivor (A93) (Table 1).

The frequency and pattern of relapse differed considerably in patients with ET, PLAGL2. Relapses/progressions occurred more often, earlier and involved more frequently distant leptomeningeal and intraventricular sites. Four of the eight first relapses or progresses occurred locally, while 3/8 occurred at distant sites and 1/8 at local and distant sites concurrently. Subsequent relapses or progress ultimately involved distant sites in all relapsed patients. As described above, relapse/progression occurred in two patients before the onset of treatment, with response to treatment and continuous remission thereafter. Five first relapses or progressions occurred during or within 3 months after the end of primary treatment, and one occurred after 17 months of remission. All six patients with relapse/progression during or after treatment ultimately succumbed to their disease. Four of the six patients received relapse treatment with additional surgery, chemotherapy and, in three cases, additional radiotherapy (one CSI/boost, one local radiotherapy and one CSI/boost and later local reirradiation). For those four patients with relapse treatment, the median OS after the diagnosis of first relapse was 3.0 years (range 2.4–3.2 years). The two patients who did not receive relapse treatment succumbed to their disease within 4.5 months after the end of their primary therapy. From the additional cohort of patients with incomplete clinical data, a further patient with ET, PLAGL2 (A110), who received a complete macroscopic resection and was treated with induction/HDCT/ASCR (no radiotherapy), was alive without evidence of disease after 2.25 years at last follow‐up (Table 1).

### 3.8 Survival Analysis

**FIGURE 3:**
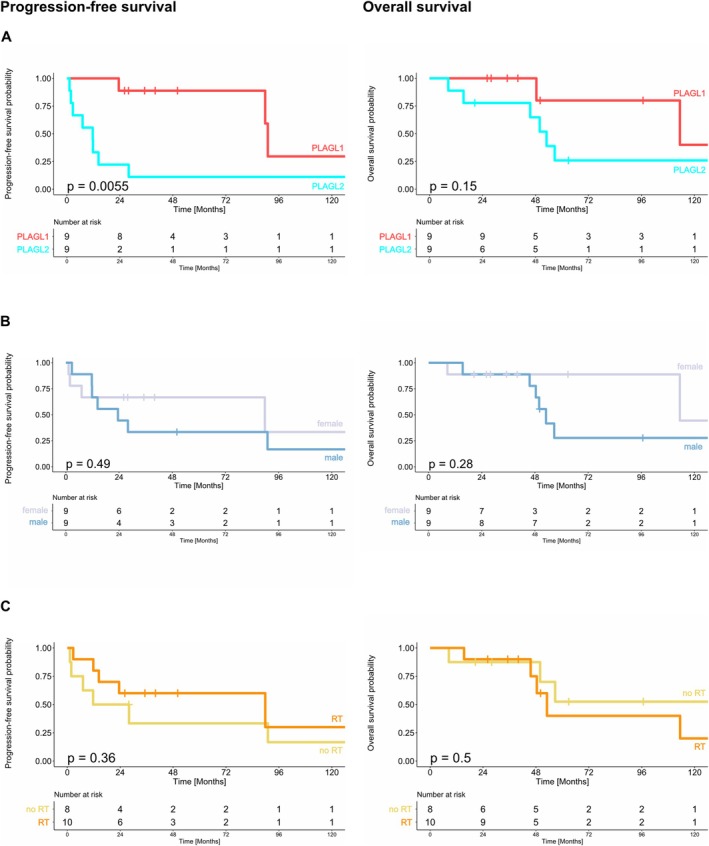
Kaplan–Meier plots showing 10‐year OS and PFS stratified by (A) subtype, (B) sex and (C) application of radiotherapy during primary treatment. The log‐rank test was used to show differences between the curves, *p* values of the log‐rank test are shown in each graph.

No association was observed between the resection status or supratentorial/infratentorial tumour localisation and survival (Fisher's exact test, *p* = 0.6372 and 0.3665, respectively). Six of the 10 survivors started therapy with an incompletely resected tumour but achieved a complete remission over the course of the treatment (Figure 1). Similarly, the tumours of survivors and deceased patients were distributed across various locations, that is, supratentorial hemispheric, supratentorial midline, infratentorial cerebellar‐hemispheric and infratentorial brainstem (Figure 2A). PFS and OS were determined for all 18 patients stratified by tumour subtype, sex and inclusion of radiotherapy during primary treatment (Figure 3). PFS differed significantly between patients with ET, PLAGL1 and ET, PLAGL2 (Figure 3A) (log‐rank test, *p* = 0.0055), but not between male and female patients (Figure 3B) (log‐rank test, *p* = 0.49), or patients who received or did not receive radiotherapy during primary treatment (Figure 3C) (log‐rank test, *p* = 0.36). No clear difference was seen between the OS curves of ET, PLAGL1 and ET, PLAGL2 (log‐rank test, *p* = 0.15), though a trend towards better OS of patients with ET, PLAGL1 compared to patients with ET, PLAGL2 was noted (Figure 3A). Similarly, there was no difference in OS between female and male patients (log‐rank test, *p* = 0.16) or for the initial use of treatment regimens that include radiotherapy (*n* = 10) compared with the initial use of radiotherapy‐avoiding regimens (*n* = 8) (log‐rank test, *p* = 0.64) (Figure 3B,C).

## 4 Discussion

DNA methylation‐based CNS tumour classification has become an important tool in brain tumour diagnostics over recent years and was acknowledged as such in the latest WHO classification of CNS tumours [1, 4, 5]. Furthermore, its application has led to the identification and subsequent molecular and clinical characterisation of novel rare tumour types that would otherwise have been challenging to identify [9, 10, 11, 17]. One such tumour type is the CNS embryonal tumour with PLAGL amplification, characterised by amplification of either *PLAGL1* or *PLAGL2*, which was recently described by our group [10]. Besides reported similarities of *PLAGL1*‐ and *PLAGL2*‐amplified tumours with respect to gene expression, histomorphology and histopathology as well as a clear segregation of all ET, PLAGL tumours from other tumour types by methylation data derived *t*‐SNE analysis, ET, PLAGL1 and ET, PLAGL2 were reported to show subtle epigenetic differences suggestive of two different tumour subtypes. Despite the comprehensive molecular description of ET, PLAGL, information on the available effective therapy options or further potential clinical differences between ET, PLAGL1 and ET, PLAGL2, besides the difference in age, were previously not investigated and remain elusive. This creates a clinical dilemma: While it is now possible to classify precisely this new tumour type, there is no evidence to guide the necessary treatment decisions.

In the absence of therapeutic evidence, it is a medical need and ethical obligation to document and describe the applied treatment [18]. To address this need, we analysed extensive clinical and imaging information on patients with ET, PLAGL to identify potentially effective treatment strategies for this rare tumour type.

We collected detailed clinical and imaging data on 18 patients with ET, PLAGL, nine patients each with ET, PLAGL1 and ET, PLAGL2. This is, at the current point in time, the largest cohort of clinically annotated patients with this newly defined tumour type. To date, only single‐case reports of other clinically annotated cases have been published [19, 20].

Due to the heterogeneous presentation, the variety of historical diagnoses and the absence of therapeutic standards, the applied treatments were unavoidably highly heterogeneous.

As described in our previous study, there were relevant differences in the clinical presentation between patients with ET, PLAGL1, who were older and more often female as opposed to patients with ET, PLAGL2, who tended to be younger and more often male [10]. We further showed that both ET, PLAGL1 and ET, PLAGL2 can arise at various supratentorial and infratentorial locations, including relatively frequent occurrence in supratentorial midline structures and the brainstem, and have a propensity for developing metastases at initial presentation and/or relapse [15]. All primary tumours were located intra‐axially, and all metastases occurred in the intraventricular or leptomeningeal compartment. No clear association between the location of the initial tumour and the extent of resection was observed, but a complete resection was more often achieved for patients with ET, PLAGL1 as compared to patients with ET, PLAGL2.

We observed a discrepancy between the treatment given to ET, PLAGL1 and ET, PLAGL2 patients, partly as a function of the different age spectrums and partly as a consequence of a more aggressive clinical presentation of ET, PLAGL2 with pretreatment progression. Most patients with ET, PLAGL1 in our series were older than 3 years at diagnosis and received radiotherapy within the primary treatment, often combined with different chemotherapy regimens. Patients with ET, PLAGL1 who were younger than 3 years at diagnosis were treated with chemotherapy alone in the primary treatment, and none received high‐dose chemotherapy with autologous stem cell rescue. All patients with ET, PLAGL1 achieved a complete remission in the course of the primary treatment—irrespective of initial resection status, which was not associated with survival probability. The observed responses to chemotherapy in two patients and to chemotherapy and subsequent radiotherapy in two further patients suggest chemosensitivity of this tumour type, while no definitive conclusions can be drawn about its radiosensitivity. Three of nine patients experienced relapses, which occurred late and were independent of the use of initial radiotherapy. While acknowledging the risk of overinterpretation, it is noteworthy that the only patient with metastatic relapse had been treated with initial local irradiation alone, while no metastatic relapses were reported after combined treatment with chemotherapy and local radiotherapy. Although our data indicate the propensity for this tumour type to develop metastases at initial presentation and relapse, the limited data do not currently support the necessity of craniospinal irradiation for patients with localised ET, PLAGL1.

The majority of patients with ET, PLAGL2 were younger than 3 years at presentation and received different types of chemotherapy. Two patients experienced rapid tumour progression before therapy started, illustrating the potential disparities in growth pattern and rate between ET, PLAGL1 and ET, PLAGL2, as such rapid regrowth was not observed in ET, PLAGL1. Similar to ET, PLAGL1, responses to chemotherapy were observed both before radiotherapy and after subsequent radiotherapy. Relapse of ET, PLAGL2 occurred early in most patients, and half of the first and all of the subsequent relapses were distant. They occurred in radiotherapy‐naïve patients and after local radiotherapy. Only three of nine patients with ET, PLAGL2 remained in remission in the observation period. All three had pretreatment residual tumours, which did not adversely affect the outcome. Furthermore, all three were treated with high‐dose chemotherapy and autologous stem cell rescue. Two of these patients subsequently did not receive radiotherapy, while one patient received low‐dose craniospinal irradiation with a boost to the tumour bed. Despite the small case numbers, the data may indicate a benefit from intensified chemotherapy. The aggressive behaviour and high rate of early distant relapses in ET, PLAGL2 speak against the use of local irradiation. It remains to be clarified whether an intensified chemotherapy approach alone may be sufficient for disease control or if subsequent craniospinal irradiation, possibly at a low dose, may be beneficial for patients old enough to tolerate this. Of note, in this cohort, none of the patients who relapsed on or after treatment could be salvaged, regardless of whether craniospinal irradiation was used in the relapse treatment.

We have previously defined ET, PLAGL as an embryonal neoplasm based on its histological and immunohistochemical features, namely, primitive embryonal‐like cells with high mitotic activity in the concurrent absence of glial or neuronal differentiation markers, as well as a gene expression profile indicative of an early developmental state [10]. We show in this study that ET, PLAGL also display clinical behaviour comparable to embryonal tumours with both primary intra‐axial tumours and intraventricular and leptomeningeal but no extradural metastases. We, therefore, suggest staging as introduced by Chang et al. for embryonal tumours [21]. However, the description of this idiosyncratic tumour type as an embryonal tumour does not mean that treatment standards from other embryonal tumours, such as medulloblastoma or ATRT, are the most appropriate. In particular, the necessity to use craniospinal irradiation, a highly toxic treatment modality with an impact on life‐long health [22], needs to be scrutinised.

Our data suggest that the epigenetically distinct ET, PLAGL1 and ET, PLAGL2 represent clinically distinct subtypes of ET, PLAGL that show differences in growth and relapse pattern as well as survival outcome. For the more common embryonal tumours, such as medulloblastoma and ATRT, the clinical relevance of molecular subtypes has long been established [23, 24, 25]. Type‐specific heterogeneity may, therefore, also be relevant for the new, molecularly defined embryonal tumour types like ET, PLAGL. As with other recently identified tumour types, clinical data are needed to establish tumour type‐ and subtype‐specific treatment recommendations [26, 27]. The clinical differences between ET, PLAGL1 and ET, PLAGL2 in this study need further validation in larger cohorts as the small numbers preclude deducing formalised treatment recommendations. Given, however, the rarity of the tumour type and the severity of the disease as well as the lack of standardised treatment recommendations for newly discovered, molecularly defined malignant tumour types such as ET, PLAGL [27], the data presented here may offer preliminary insights for potential treatment decisions.

Our data show that precise diagnosis is of considerable clinical relevance. Additional adjuvant therapy, beyond surgery alone, should be applied in all patients with ET, PLAGL independent of resection status, while the optimal treatment modalities are likely to be different for ET, PLAGL1 and ET, PLAGL2. Patients with ET, PLAGL2 might benefit from HDCT with ASCR, but not from local radiotherapy, whereas patients with ET, PLAGL1 might be effectively treated with less intensive chemotherapy regimens, with or without local radiotherapy for their primary disease, and should be monitored for late recurrences.

Despite the rarity of this tumour type and the small size of our cohort, the integrated analysis of clinical information and MRI data provides valuable insights into this rare disease group. While the small number of cases and the heterogeneity of treatments are limiting factors, our findings contribute significantly to the understanding of ET, PLAGL. Due to the rarity of the tumour type and the current lack of evidence, it is crucial to document the presentation, treatment and outcomes for patients with ET, PLAGL and collect this information in combination with molecular data. When expanded upon within broad international collaborations, these data may be used for developing treatment standards and prospective clinical trials. Beyond international collaborative clinical and molecular data collections, international tumour boards may directly support the treating oncologists and also contribute to the stepwise process of developing more detailed treatment recommendations.

In our initial publication on ET, PLAGL, we reported the general absence of recurrent genetic alterations by next‐generation DNA sequencing (NGS), with the amplification and subsequent overexpression of *PLAGL1* or *PLAGL2* as the only recurrent molecular event detected in these tumours, together with the overexpression of candidate drug targets such as *RET* and *CYP2W1* [10]. We also reported coamplification of *CBFA2T2* in a subset of *PLAGL2*‐amplified tumours, which we also screened for in this cohort, however, with no clinically meaningful correlations (data not shown). Further analyses on the molecular spectrum and its clinical relevance, as well as (sub)type‐specific biomarkers, will need to be performed and evaluated in larger cohorts in the future. Identifying targetable alterations and potentially effective new drugs using patient‐derived or genetically engineered models will be of utmost importance.

## Author Contributions

Conception and design, original draft writing, review and editing: M.‐K.K., A.T., K.v.H. and D.T.W.J. Material preparation and data collection, manuscript review and editing: all authors. Data analysis: A.T., B.B., M.‐K.K. and K.v.H. Data interpretation: A.T., M.‐K.K. and K.v.H.

## Ethics Statement

Core study data were collected within the MNP2.0 study in accordance with the respective ethics approval (S‐320/2014; Ethics Committee of the Medical Faculty of Heidelberg University). Additional study data were collected by the respective collaborating national or international institutions in compliance with relevant local ethics and data privacy regulations, including patient/parental consent as appropriate.

## Conflicts of Interest

The corresponding authors report no potential conflict of interest. Per Nyman reports minority private shareholding (SyntheticMR AB). Laura S. Korhonen is a NOPHO (Nordic Society of Paediatric Haematology) board member. Pieter Wesseling received a travel grant from Chimerix for contributing to a session during the Annual SNO 2023 meeting in Vancouver and is chair of the cIMPACT‐NOW Steering Committee. Ingrid Øra is supported by the Swedish Childhood Cancer Fund (Stockholm, Sweden). Christof M. Kramm is supported by the Deutsche Kinderkrebsstiftung (Bonn, Germany) and has received grants/contracts from Blueprint Rover and Novartis. He is also on the advisory board on glioblastoma medication (Boehringer Ingelheim), chairman of the HIT‐HGG study group, (Goettingen, Germany), executive committee member of the SIOPE DIPG Registry, Utrecht, the Netherlands, and executive committee member of the Neurooncological Working Group (Berlin, Germany). Juhana Hakumäki is president of the Finnish Radiological Society. Michal Zapotocky has received an honorarium from AstraZeneca. Where authors are identified as personnel of the IARC/WHO, the authors alone are responsible for the views expressed in this article and they do not necessarily represent the decisions, policies or views of the IARC/WHO.

## Supporting information


**Data S1.** Supporting Information.

## Data Availability

Further details of the respective treatment protocols can be made available upon request.
